# Impact of socio-economic and political factors on global COVID-19 vaccine coverage: an empirical study

**DOI:** 10.1186/s41182-025-00877-4

**Published:** 2026-01-05

**Authors:** Duc Hoang Nguyen, Linh Tran, Nguyen Truong Vien, Mohammad Rashidul Hashan, Ashesh Tripathi, Su Myat Han, Anh Hoang Nguyen, Ngo Binh Trinh, Eithar Elias Shabbo, Dang Xuan Thang, Pham Le An, Gladson Vaghela, Nguyen Tien Huy

**Affiliations:** 1 Hanoi Medical University, 1 Ton That Tung, Hanoi, Vietnam; 2 Cardiovascular Laboratory, Methodist Hospital, Merrillville, IN USA; 3 Online Research Club, Nagasaki, Japan; 4 Faculty of Pharmacy, University of Health Sciences, Vietnam National University Ho Chi Minh City, Ho Chi Minh City, Vietnam; 5 Pham Ngoc Thach University of Medicine, Ho Chi Minh City, Vietnam; 6 School of Medical, Health and Applied Sciences, Central Queensland University, Rockhampton, Australia; 7 Directorate General Health Services, Ministry of Health and Family Welfare, Government of Bangladesh, Dhaka, Bangladesh; 8 School of Tropical Medicine and Global Health, Nagasaki University, Nagasaki, Japan; 9 Department of Infectious Disease Epidemiology, Faculty of Epidemiology and Population Health, London School of Hygiene and Tropical Medicine, London, UK; 10 Thai Binh University of Medicine and Pharmacy, Thai Binh City, Vietnam; 11 Department of Dermatology, University of Medicine and Pharmacy at Ho Chi Minh City, Ho Chi Minh City, Vietnam; 12 Ahfad University for Women, School of Medicine, Khartoum, Sudan; 13 Institute of Research and Development, Duy Tan University, Da Nang, Vietnam; 14 School of Medicine and Pharmacy, Duy Tan University, Da Nang, Vietnam; 15 Family Medicine Training Center, University of Medicine and Pharmacy at Ho Chi Minh City, Ho Chi Minh City, Vietnam; 16 Nuffield Department of Population Health, University of Oxford, Oxford, UK; 17 Oxford India Centre for Sustainable Development, University of Oxford, Oxford, UK; 18 Research Center for Discovery and Development of Healthcare Products, Vietnam National University Ho Chi Minh City, Ho Chi Minh City, Vietnam; 19 Cardiology Department, Ha Nam General Hospital, Ninh Binh, Vietnam; 20 Department of General Surgery, Institute of Medicine, Tribhuvan University, Kathmandu, Nepal

**Keywords:** COVID-19, Vaccination, Vaccine coverage, Socio-demographic factors, Impact

## Abstract

**Purpose:**

This study seeks to explore the COVID-19 vaccine coverage across various countries by delving into its connections to seven vital indicators, these include the Human Development Index (HDI), Gross National Income (GNI) per capita, health expenditure, Internet usage, political stability, absence of violence and their correlation with the vaccine coverage.

**Measures:**

This study utilized a wealth of information from three valuable and publicly accessible data sources, such as Our World in Data, the World Bank, and the WHO database as of 20 March 2023. We then employed correlation analysis, linear regression, and structural equation modeling to examine the intricate relationships between various indicators and vaccine coverage, illuminating patterns at both national and continental levels.

**Results:**

Our comprehensive research unveiled that on an average countries around the world achieved a 54.5 ± 24.61% of COVID-19 vaccine coverage rate. Six of the seven indicators emerged to have positive correlation with the COVID-19 vaccine coverage, and they are the HDI, individuals using the internet, current health expenditure, political stability and absence of violence/terrorism, total cases per million people, and the total deaths per million people. Among these, HDI stood out as the strongest correlated indicator, and conversely, the percentage of rural population emerged as a negatively correlated indicator in relation to the vaccine coverage.

**Conclusions:**

These findings illuminate the formidable challenges associated with the quest for achieving universal vaccine coverage. In the future to address various pandemics globally, these insights emphasize the critical need for developing targeted strategies, fostering international collaboration and implementing comprehensive approaches to ensure that vaccines are fairly and equitably distributed and ultimately foster global immunity.

**Supplementary Information:**

The online version contains supplementary material available at 10.1186/s41182-025-00877-4.

## Introduction

Vaccine equity denotes all aspects of the vaccine process. This includes supporting all those with greater needs, minimizing disparities, and addressing both the short-term disease containment and underlying structural injustices for long-term repair [1]. Global Equity is one of the six cornerstone values of the WHO’s Strategic Advisory Group of Experts (SAGE’s) ethical framework for global vaccine allocation [2].

On March 11th, 2020, the WHO declared a global Pandemic. The urgent need to develop COVID-19 vaccines and their unprejudiced distribution was the vital strategy for combating and controlling the crisis [3, 4]. Carrying out a robust and widespread vaccination campaign will help safeguard people's health and welfare, especially when the proportion of individuals vaccinated surpasses 60% [5]. As reported by WHO, most of the COVID-19 vaccines have been administered in high- and upper-middle-income countries [6]. The COVID-19 vaccines global access (COVAX) facility served as a beacon of hope during the challenging time to ensure global equity [7]. It aimed at providing significant guidance globally in achieving equitable access to vaccines and supported the vaccine procurement and distribution to the low- and middle-income countries (LMICs) [8]

Denoting global COVID-19 vaccine inequality, in the low-income countries, only 32.6% of the population received at least one dose of the COVID-19 vaccine [9]. Inadequate supply, unfair distribution, limited vaccine production in low-income nations, fragile healthcare systems, vaccine hesitancy, and misunderstandings about vaccines are the primary causes for the differences in vaccine coverage [10]. In addition to the ethical shortcomings, the unfairness and the unjust vaccine distribution threaten the economic and human health negatively and impede the achievement of global population immunity, as COVID-19 has no borders in transmission. As per previous modeling studies, 50% of the deaths could have been prevented in the low- and middle-income countries if they had a similar vaccination rate to that of the high-income countries. Moreover, if they had at least a similar early access as the high-income countries, there could have been a significant decrease in the percentage of deaths (range 6–50%) [11]. Studies have suggested that if low- and middle-income countries had much stricter and extended public health measures, the number of deaths that could have been averted would have been equivalent to the numbers observed in the scenarios with high vaccine availability [12, 13]. According to the WHO director, the disparities in the vaccine distribution are not just a moral lapse but also “an epidemiological failure, which is creating the ideal conditions for new variants to emerge” [14].

Socio-economic and socio-demographic factors, such as living in crowded conditions, having jobs that require public interactions, limited access to healthcare, transport, and even language barriers, are the causes of the unequal outcomes from COVID-19 infection in low- and middle-income countries(LMICs) [15]. Limited or no internet access in some regions of the LMICs could be another factor that could cause difficulties in making online vaccine appointments [10].

In the beginning stages of the pandemic, some of the studies conducted displayed that the disparities in the vaccine distribution were due to a positive correlation between the vaccination and the socio-demographic factors, such as Human Development Index (HDI), GDP per capita, and life expectancy [14]. Similarly, there was a positive correlation in countries with higher HDI and living Gross National Income (GNI) per capita and vaccination rates, as these were the countries that were given preference for vaccine access [15]. One of the studies conducted attributed that due to the four factors: level of human development, COVID-19 fatalities, the GDP per capita, and population density, there was a 50% variation in the number of days a country received vaccination [16]. The Vaccine Rollout Index (VRI) is an important metric that captures the swiftness and the scope of vaccination efforts across the nation’s population. This index is closely tied to the four critical factors—the median per capita income, HDI, percentage of individuals who accessed the internet in the past 3 months, and per capita health expenditure in understanding the effectiveness of the vaccination programs [17].

This research delves into the intricate details of the global vaccination coverage during the later phases of the COVID-19 pandemic, and it highlights the presence of glaring inequalities that plague the vaccination efforts across different countries. We thoroughly analyzed the correlation between the various socio-demographic factors, such as the HDI, current health expenditure, internet usage, political stability, and absence of violence, rural population, total cases per million, total deaths per million, and the vaccine uptake. Through this study, we aim to provide valuable insights into the multi-faceted challenges in the landscape of global vaccination efforts. Ultimately, with these findings, we aim to empower healthcare workers and policymakers with strategies to enhance vaccine accessibility and to bridge gaps across diverse populations worldwide.

## Methods

### Data sources and study design

Data were sourced from three publicly accessible repositories: our world in data COVID-19 [18], world bank [19], and the WHO database [20] as of March 20, 2023. A total of 186 countries was included in the analysis (Supplemental Fig. 1). We included countries in this study based on the availability of a COVID-19 vaccination coverage index and recognition by the Statistic Times' continental categorization. In addition, countries reporting vaccination rate exceeding 100% were excluded.

We selected seven indicators to examine their relationships with COVID-19 vaccine coverage: human development indicator (HDI) [21], Internet usage [22], current health expenditure per capita [23], percentage of rural population [19], political stability and absence of violence/terrorism: estimate [24], total cases per million and total deaths per million [25]. These seven indicators were considered as the most suitable and relevant to the outcome of the study based on data availability and reliability. (Supplemental Table 1).

### Statistical analysis

The median, interquartile range (IQR), mean, and standard deviation (SD) were used to express all quantitative variables in the descriptive analysis. Pearson correlation and linear regression were used to express the linear relationships between variables in univariable analysis. In addition Spearman correlations were added as a sensitivity analysis to assess the effect of non-normality distribution assumption violations on linear relationships. The results of Pearson correlations were found to be reliable, because it showed similarity to other results Spearman correlation.

We used the coefficient of determination (R2) to assess the goodness of fit at the equation level. The degree and direction of correlations between variables were described using correlation coefficients, which have a range of − 1 to + 1. We employed structural equation modeling (SEM), a statistical technique that enables the simultaneous study of several variables and pathways, to investigate the relationships inside our conceptual model. When evaluating complex models with latent variables and both direct and indirect effects, SEM is especially helpful. SEM was used in this investigation to evaluate the direct and indirect relationships outlined in our framework. The results of the SEM analysis are reported as correlation coefficients, along with 95% confidence intervals and *p* values. A *p* value of less than 0.05 was considered statistically significant. All analyses were conducted using Stata version 16.1.

## Results

### Characteristics of the collected data and variables

We analyzed 186 countries in total (Supplemental Fig. 1). The descriptive statistics of socio-economic variables and COVID-19 vaccine coverage are summarized in Table 1. The mean COVID-19 vaccine coverage was 54.59% (SD =  ± 24.61%), while its mean was 56.56% (IQR 35.29–73.90%). In line with this, the average HDI was 0.72 (SD =  ± 0.15), with a median of 0.731 (IQR 0.596–0.832), and its range was from 0.38 to 0.96.

**Table 1:** Characteristics of socio-economic variables and COVID-19 vaccine coverage (*n* = 186)

	Mean ± SD	Median (Quartile)	Range
COVID-19 vaccine coverage	54.59 ± 24.61	56.56 (35.29–73.90)	0.00–99.01
HDI	0.72 ± 0.15	0.731 (0.596–0.832)	0.38–0.96
Individuals using the Internet (% of population)	60.43 ± 28.58	70 (35–86)	1–100
Current health expenditure (% of GDP)	6.54 ± 2.92	6.21 (4.44–8.04)	1.80–23.96
Rural population (% of total population)	40.76 ± 22.74	41 (22–59)	0.00–87
Political Stability and Absence of Violence/Terrorism	(− 0.95) ± 0.96	− 0.109 (− 0.670 to 0.756)	(− 2.66)–1.63
Total cases per million	160,563 ± 180,963	91,898 (11,703–245,169)	0–729,533
Total deaths per million	1248 ± 1347	752 (130–2000)	0–6454

Internet accessibility was another variable of interest, with a median of 70% (IQR 35–86%), average of 60.43% (SD =  ± 28.58%), and range of 1–100%. The large SD indicated a broad disparity in online access across nations. The results also reflected differences in healthcare expenditure, with countries allocating an average of 6.54% (SD =  ± 2.92%) of their GDP to healthcare. The median was 6.21% (IQR 4.44–8.04%) and the range was from 1.80% to 23.96%. An average of 40.76% (SD =  ± 22.74%) of the population studied was rural, which is close to the median of 41% (IQR 22–59%) and the range is 0–87%.

The political climate also varied widely across countries. The average score for political stability and absence of violence or terrorism was − 0.95 (SD =  ± 0.96), while the median was − 0.109 (IQR = − 0.670 to 0.756), signifying a general trend of political instability, and values ranged between − 2.66 and 1.63. As for the pandemic's toll, the median number of COVID-19 cases per million stood at 91,898 (IQR 11,703–245,169), but the mean was significantly higher at 160,563 (SD =  ± 180,963), with the range being 0–729,533. The average mortality rate was 1248 (SD =  ± 1347) deaths per million, yet some nations reported zero deaths, while others faced as many as 6454 deaths per million.

### Correlation between COVID-19 vaccine coverage with each indicator—univariate analysis

In our analysis, out of the seven variables examined, six were positively correlated with COVID-19 vaccine coverage, while the proportion of rural population demonstrated a negative correlation. The correlation matrix is shown in Supplemental Fig. 2. Detailed examination of the correlations provided insights into the specific magnitude and implications of each variable's influence on COVID-19 vaccine coverage across the 186 studied countries (Table 2).

**Table 2:** Correlation between COVID-19 vaccine coverage with each indicator—univariable analysis (*n* = 186)

COVID-19 vaccine coverage	Spearman’s correlation		Pearson correlation		Linear regression	
	*R*	*p*	*R*	*p*	Coef	*R*^2^ (%)
HDI	0.601	< 0.001	0.620	< 0.001	101.340 (82.702–119.987)	38.47
Individuals using the Internet (% of population)	0.572	< 0.001	0.574	< 0.001	0.494 (0.392–0.597)	32.93
Current health expenditure (% of GDP)	0.334	< 0.001	0.327	< 0.001	2.750 (1.593–3.907)	10.67
Rural population (% of total population)	− 0.408	< 0.001	− 0.401	< 0.001	− 0.434 (− 0.578 to − 0.290)	16.06
Political stability and absence of violence/terrorism	0.517	< 0.001	0.537	< 0.001	13.701 (10.570–16.833)	28.83
Total cases per million	0.549	< 0.001	0.476	< 0.001	0.00006 (0.00004–0.00008)	22.63
Total deaths per million	0.286	< 0.001	0.195	< 0.001	0.0034 (0.0009–0.0060)	3.80

R^2^: coefficient of determination

**Fig. 1:**
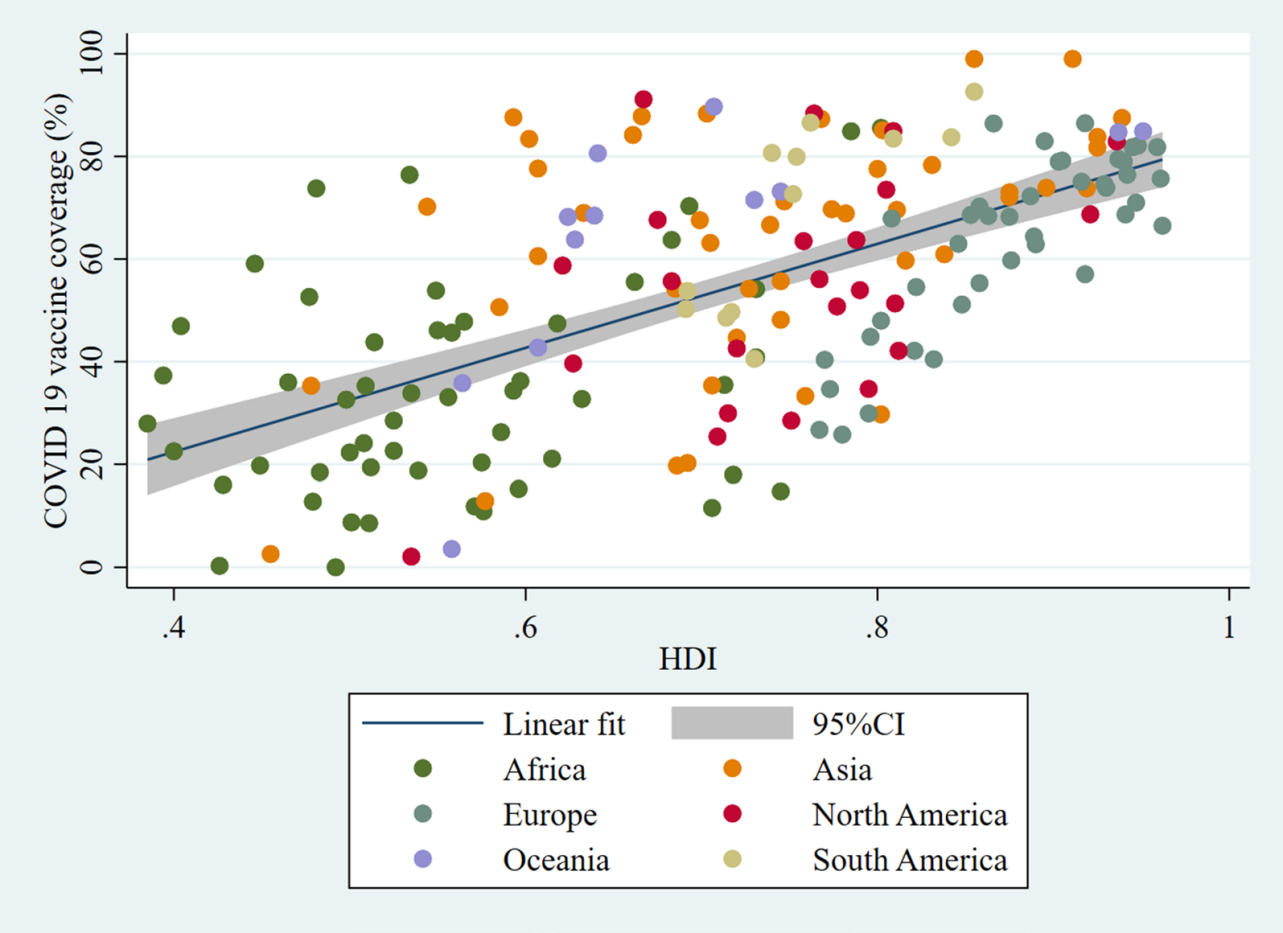
Correlation graph between COVID-19 vaccine coverage and HDI. Created using: Stata version 16.1

HDI showed the strongest positive correlation with COVID-19 vaccine coverage out of the seven studied socio-economic variables. Both Spearman’s and Pearson correlation values were statistically significant at 0.601 and 0.620, respectively, with *p* < 0.001. The linear regression model showed that for every unit increase in HDI, the COVID-19 vaccine coverage tends to increase by approximately 38.47% (Fig. 1).

Internet access also demonstrated a notable positive correlation with COVID-19 vaccine coverage. Spearman's correlation coefficient was 0.572, and Pearson's was 0.574, both significant at *p* < 0.001. The linear regression revealed an expected increase in vaccine coverage of 0.494% for every 1% rise in internet usage, explaining about 32.93% of the variance. This shows that for every additional 1% of the population that has internet access, there is approximately a 0.5% increase in COVID-19 vaccination rate. This relationship suggests that internet access may account for about one-thirds of the differences we see in vaccination rates across countries (Supplemental Fig. 3).

Current healthcare expenditure as a percentage of GDP, although positively correlated with vaccine coverage, had a moderate strength of association. The Spearman’s and Pearson’s correlation values were 0.334 (*p* < 0.001) and 0.327 (*p* < 0.001), respectively. The regression model suggested that for every 1% increase in health expenditure, vaccine coverage increases by about 2.75%, which accounts for 10.67% of the variability in coverage (Supplemental Fig. 4).

In contrast, the percentage of the rural population showed a negative correlation with vaccine coverage, with Spearman's and Pearson's coefficients being − 0.408 (*p* < 0.001) and − 0.401 (*p* < 0.001), respectively. The regression model indicates a 0.434% decrease in vaccine coverage for every 1% increase in the rural population, explaining 16.06% of the variance (Supplemental Fig. 5).

Political stability and the absence of violence and terrorism was also positively correlated with COVID-19 vaccine coverage. The Spearman’s correlation was 0.517 (*p* < 0.001) and Pearson's correlation was 0.537 (*p* < 0.001). The regression model showed that for every 1% enhancement in political stability, vaccine coverage increased by 13.701%, explaining 28.83% of the variance (Supplemental Fig. 6).

Finally, the total cases of COVID-19 and number of COVID-19-related deaths per million each demonstrated a positive relationship with vaccine coverage. For the total number of cases per million, Spearman's and Pearson's values were 0.549 (*p* < 0.001) and 0.476 (*p* < 0.001), respectively, while for total deaths per million, these values were comparatively lower at 0.286 (*p* < 0.001) and 0.195 (*p* < 0.001). The regression models indicated small but statistically significant coefficients for both variables, accounting for 22.63% and 3.80% of the variance in vaccine coverage, respectively (Supplemental Figs. 7 and 8).

### COVID-19 vaccine coverage in the context of national capacity and impact

**Fig. 2:**
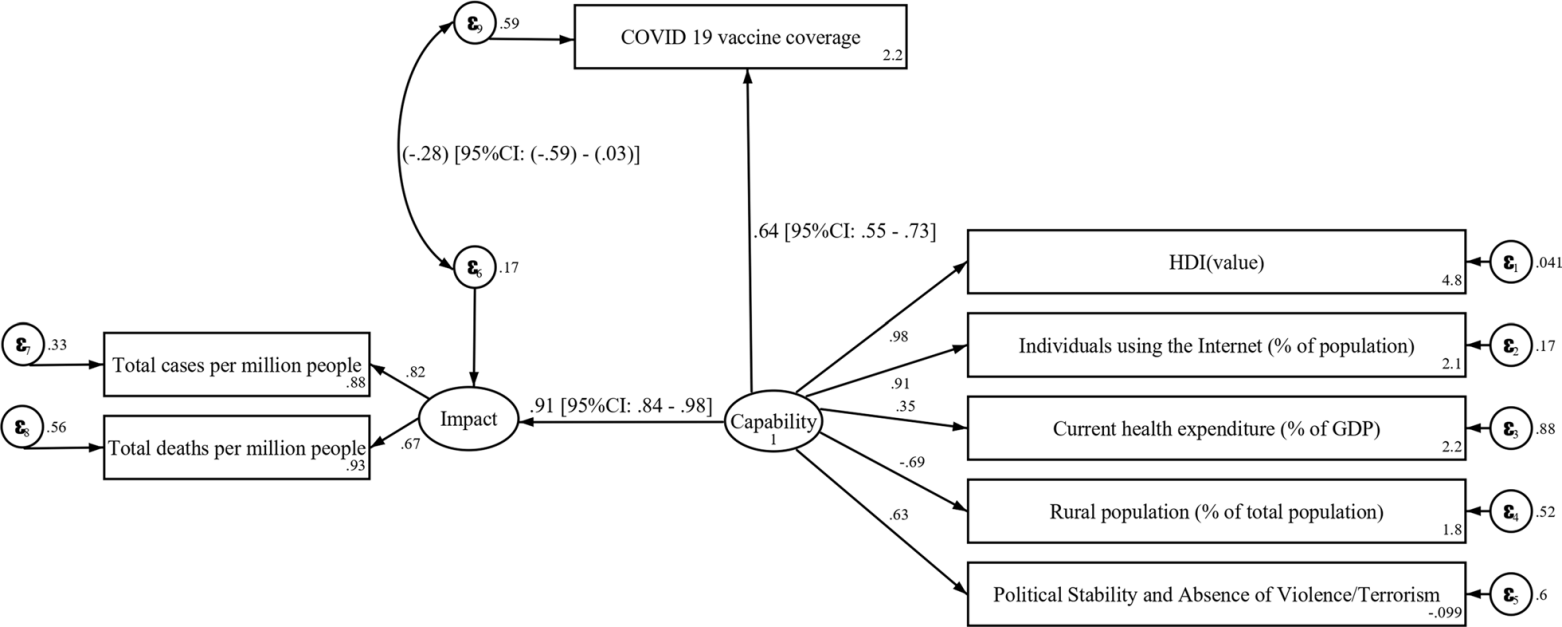
Structure model for vaccine coverage interpretation in the context of national capacity and impact (*n* = 186). Created using: Stata version 16.1

This study offers several significant insights into examining COVID-19 vaccine coverage in relation to national capacity and impact (Fig. 2). The association between vaccine coverage and its impact on the population presented a value of − 0.28 (95% CI − 0.59 to 0.03, *p* = 0.051). A strong positive relationship emerged when assessing the coefficient between vaccine coverage and national capability. With a standardized estimate of 0.64 (95% CI 0.55–0.73, *p* < 0.001), the results indicate that as a nation's capability improves, its vaccine coverage also increases (*p* < 0.001). This study observed a strong positive relation between the impact and national capability with high coefficient 0.91 (95% CI 0.84–0.98, *p* < 0.001), indicating that the countries with greater capabilities generally experience higher impacts.

The relation between the impact with total cases per million is 0.82 (95% CI 0.74–0.90) and with total deaths per million is 0.67 (95% CI 0.57–0.76). Both these associations are statistically significant, indicating a strong relationship between the impacts of the pandemic and the total number of cases and deaths per million people.

In terms of socio-demographic factors, the HDI, internet usage, and political stability and absence of violence/terrorism possess statistically strong positive coefficients with values of 0.98, 0.91, and 0.63, respectively. In addition, the current health expenditure's relationship with capability provides a coefficient of 0.35, indicating a moderate positive association. Notably, the relation between the rural population and capability is negative, with a coefficient of − 0.69 (95% CI − 0.77 to − 0.61), suggesting that when the percentage of rural population increases, the national capability probably reduces.

### Equation-level goodness of fit

The overall model demonstrates strong predictive capability with an *R*^*2*^ value of 0.934, indicating that 93.4% of the variance in the dependent variable can be explained by the model’s predictors. Analysis of individual dependent variables, however, showed a wide variance in *R*^*2*^ values. Vaccine coverage has an R^2^ of 0.406, accounting for 40.6% of its variance among the countries analyzed, whereas HDI is considerably better explained with *R*^*2*^ of 0.959 capturing 95.9% of its variance across countries (Table 3, equation-level goodness of fit).

**Table 3:** Equation-level goodness of fit (*n* = 186)

	R^2^
Overall model	0.934
Dependent variables	
Vaccine coverage	0.406
Impact	0.835
HDI	0.959
Individuals using the internet (% of population)	0.834
Current health expenditure (% of GDP)	0.123
Rural population (% of total population)	0.480
Political stability and absence of violence/terrorism	0.397
Total cases per million	0.672
Total deaths per million	0.444

R^2^: Coefficient of determination

For technology access, the percentage of individuals using the internet showed an *R*^*2*^ value of 0.834, suggesting the model can elucidate roughly 83.4% of the variance observed in internet usage rates. However, the model's predictive power was relatively low for the current health expenditure (% of GDP), with an *R*^*2*^ value of only 0.123, indicating the model can explain just about 12.3% of the variance in this metric. Similarly, other metrics such as the rural population, political stability, total cases per million, and total deaths per million yielded *R*^*2*^ values of 0.480, 0.397, 0.672, and 0.444, respectively.

## Discussion

The impact of the pandemic has been a global phenomenon; however, the distribution and implementation of vaccination varied significantly among nations. This study embarked on a comprehensive analysis to decipher these disparities. Our results provide a mosaic of insights that paint a coherent picture, but it becomes even more intriguing when juxtaposed with findings from existing research.

A salient observation in this study was the correlation between HDI and vaccine coverage (*r* = 0.601 and *r* = 0.620 for Spearman’s and Pearson, respectively). This echoes the findings of Makki et al., who examined HDI in countries with varying levels of development, namely, Saudi Arabia (high HDI), India (medium HDI), and Sudan (low HDI). Makki et al.’s results also showed that higher HDI was associated with more acceptance and greater belief in the COVID-19 vaccine profile [26]. The same trend was observed in Peru by Al-Kassab-Córdova et al. (coefficient *r* = 0.3807, 0.4064, and 0.4435; *p* < 0.0001 for the first, second, and third COVID-19 vaccine dose, respectively) [27]. Although there is no specific explanation for the trend in the mentioned studies, it is widely believed that countries with a higher HDI have the economic strength for robust healthcare investment, facilitating efficient vaccine distribution. Higher education levels in these nations foster awareness of vaccination benefits, while their established trust in medical institutions and effective public health policies further promote vaccine uptake.

The significant correlation between internet usage and vaccine coverage was another critical discovery. The ubiquity of technology, particularly smartphones and the internet, offers an avenue for health interventions and mass communication, especially in the context of COVID-19 vaccine campaigns [28]. This study revealed a positive correlation between the percentage of individuals using the internet and COVID-19 vaccine coverage, albeit with moderate correlation coefficients (Spearman’s and Pearson, 0.572 and 0.573, *p* < 0.001, respectively). This finding contrasts with the previous study by Wilson et al. which suggested that greater internet access may lead to increased vaccine hesitancy as consequence of the proliferation of misinformation and anti-vaccination rhetoric on social media platforms [29]. Another study in India by Nair et al. showed higher distrust in immunization program in regions with higher access to the internet [30]. A possible explanation for this trend is the rampant spread of misinformation online. The internet, particularly social media platforms, has become an efficient platform to spread misinformation or fearmongering by anti-vaxxers. This might raise a concern that public health authorities and governments should prioritize digital literacy, develop targeted communication strategies, and collaborate with tech companies to combat misleading narratives and ensure that accurate, science-based information is prioritized on online platforms.

The correlation between political stability, absence of violence/terrorism, and vaccine coverage further augments the multi-faceted nature of vaccine uptake. This study identified a significant relationship between vaccine coverage and political stability, including the absence of violence/terrorism, with a coefficient of 0.63 (0.54–0.72), *p* < 0.001, Spearman's correlation 0.517 (*p* < 0.001) and Pearson's correlation 0.537 (*p* < 0.001). These findings align with another study by Baghbanzadeh et al., which reported that nations with stable political climates and minimal violence typically have better healthcare outcomes, including higher vaccination rates. However, it was also observed that some countries perceived as corrupt had lower death rates and this may be attributed to a lack of resources, delays in reporting, or deliberate under-reporting of political motive [31]. This study provides additional evidence highlighting the role of political stability in influencing health outcomes.

The current study found a negative association between the rural population percentages and vaccine coverage. Regions with higher percentages of rural populations displayed negative relationship in terms of vaccine uptake. This finding on the disparities were consistent with the observed patterns in Saelee et al.’s study tracking vaccination coverage in the USA from December 2020 to January 2022 (rural areas had first-dose coverage of 58.5%, while urban areas had 75.4%) [32]. A similar pattern with a smaller difference was also noted in Murthy et al.’s study from December 2020 to April 2021 (38.9% and 45.7% in rural and urban areas, respectively) [33]. Such patterns may be attributed to limited healthcare infrastructure in rural areas, increased vaccine skepticism, and reduced exposure to public health campaigns. The reduced connectivity in rural zones might also make them more vulnerable to misinformation. Comparing our findings to Saelee et al. and Murthy et al., it can be shown that tailored strategies are crucial to ensure equitable vaccine distribution across regions [32, 33].

Our analysis indicated a modest positive association between current health expenditure as a percentage of GDP and COVID-19 vaccine coverage. When assessing the influence of a nation’s capability on current health expenditure, the coefficient was found to be 0.35 (0.22–0.48), *p* < 0.001. These findings suggest that countries investing more in their health systems tend to have better vaccine coverage. Prior study by Roghani revealed that high GDP countries have achieved significant vaccination coverage, but global immunity requires expanding vaccine availability in low GDP nations [34]. Moreover, Utami et al.’s study reviewing the impact of economic status on COVID-19 vaccine efficacy found that developed nations had a higher vaccine efficacy due to their more substantial financial commitments to healthcare. However, Utami et al. also highlighted that while higher health expenditure often correlated with increased vaccine efficacy, the distribution and utilization of these funds played a crucial role [35]. This aligns with a study by Castillo-Zunino et al., which emphasized the significance of health expenditure in predicting successful vaccination campaigns. However, Castillo-Zunino et al. also found that in many countries, the success of vaccination campaign may not be related to health expenditure. The efficacy of vaccination campaigns often hinges on how these funds are distributed and utilized within the healthcare system [36]. For instance, if significant portions of the health budget are channeled towards non-immunization activities, such as infrastructural developments, administrative costs, or treatment of non-communicable diseases, the impact on vaccine distribution might be minimal. Furthermore, efficient vaccination campaigns require not just funds, but also robust logistics, well-trained healthcare workers, public awareness campaigns, and community engagement. These factors partly explained the weak correlation, despite being positive, between health expenditure and vaccine coverage in this study.

Despite our findings, our research is not without limitations. This snapshot nature of our study design may not capture evolving dynamics, and while our model explains a substantial variance in vaccine coverage, other latent factors might be at play. In addition, we relied on reported national data, which can be influenced by varying data collection and reporting standards across countries, potentially introducing inconsistencies. Furthermore, our analyses primarily focused on macro-level indicators, which may overlook local disparities and nuances in vaccine coverage within nations. Cultural and socio-economic factors at the individual or community level, which could significantly influence vaccination rates, were not extensively examined. Finally, the cross-sectional nature of this study limits our ability to infer causation from observed associations, emphasizing the need for longitudinal studies in the future.

## Conclusions

This study offers a comprehensive insight of selected indicators influencing COVID-19 vaccine coverage across multiple countries. The observed associations between vaccine coverage and indicators such as HDI, internet usage, health expenditure, and political stability emphasize the interconnectedness of socio-economic and political elements in health outcomes. Furthermore, the disparities highlighted, especially in the context of rural populations, underline the persistent challenges in achieving universal vaccine coverage. As we continue grappling with the global pandemic, these insights reiterate the necessity of tailored strategies, international collaboration, and holistic approaches to ensure equitable vaccine distribution and, ultimately global immunity.

## Supplementary Information

Additional file1 (DOCX 937 KB) Description of indicators. Created using: Microsoft Office. Supplemental Fig. 1. Flowchart of country sample selection (**n = number of countries). Created using: Microsoft Office. Supplemental Fig. 2. Correlation matrix of all variables (*n* = 186). Created using: Stata version 16.1. Supplemental Fig. 3. Correlation graph between COVID-19 vaccine coverage and individuals using the internet. Created using: Stata version 16.1. Supplemental Fig. 4. Correlation graph between COVID-19 vaccine coverage and current health expenditure. Created using: Stata version 16.1. Supplemental Fig. 5. Correlation graph between COVID-19 vaccine coverage and rural population (% of total population). Created using: Stata version 16.1. Supplemental Fig. 6. Correlation graph between COVID-19 vaccine coverage and political stability and absence of violence/terrorism. Created using: Stata version 16.1. Supplemental Fig. 7. Correlation graph between COVID-19 vaccine coverage and total cases per million. Created using: Stata version 16.1. Supplemental Fig. 8. Correlation graph between COVID-19 vaccine coverage and total deaths per million.

## Data Availability

Publicly available data sets were analyzed in this study. List of indicators and their sources of data are all listed in Supplemental Table 1, which can be found in supplementary materials.
