# Supplementary material for: Impact of socio-economic and political factors on global COVID-19 vaccine coverage: an empirical study
Source: Trop Med Health. 2026 Jan 5;54:11. doi: 10.1186/s41182-025-00877-4 (PMC12781785; doi:10.1186/s41182-025-00877-4)
Supplement: Supplementary file 1 — Additional file1 (DOCX 937 KB) Description of indicators. Created using: Microsoft Office. Supplemental Fig. 1. Flowchart of country sample selection (**n = number of countries). Created using: Microsoft Office. Supplemental Fig. 2. Correlation matrix of all variables (n = 186). Created using: Stata version 16.1. Supplemental Fig. 3. Correlation graph between COVID-19 vaccine coverage and individuals using the internet. Created using: Stata version 16.1. Supplemental Fig. 4. Correlation graph between COVID-19 vaccine coverage and current health expenditure. Created using: Stata version 16.1. Supplemental Fig. 5. Correlation graph between COVID-19 vaccine coverage and rural population (% of total population). Created using: Stata version 16.1. Supplemental Fig. 6. Correlation graph between COVID-19 vaccine coverage and political stability and absence of violence/terrorism. Created using: Stata version 16.1. Supplemental Fig. 7. Correlation graph between COVID-19 vaccine coverage and total cases per million. Created using: Stata version 16.1. Supplemental Fig. 8. Correlation graph between COVID-19 vaccine coverage and total deaths per million. [file 41182_2025_877_MOESM1_ESM.docx]

**SUPPLEMENTARY RESULTS**

| **Name of indicator** | **Definition** | **Source** |
| --- | --- | --- |
| COVID-19 vaccine coverage | Cumulative number of people who completed a vaccination course per 100 population | Our World in Data^1^ |
| Human Development Index (HDI) | Summary of human development, including three main components: life expectancy, mean and expected years of schooling, and GNI per capita | Human Development Reports |
| Life expectancy at birth | The average number of years a newborn infant is expected to live | Our World in Data^1^ |
| Political stability and absence of violence/terrorism percentile rank | Measures perceptions of the likelihood of political instability and/or politically-motivated violence; percentile rank indicates the country's rank among all countries covered. | World Bank^2^ |
| Total_cases_per_million | Cumulative number of cases of COVID-19 per million population | **WHO database^3^** |
| Total_deaths_per_million | Cumulative number of deaths of COVID-19 cases per million population | **WHO database^3^** |

**Supplemental Table 1. Description of indicators**The study uses data from various sources as described in the following table.

^1^Our World in Data. Coronavirus (COVID-19) Cases [20 March 2023]. Available from: <https://ourworldindata.org/covid-cases>.

^2^World Bank. [20 March 2023]. Available from: <https://data.worldbank.org/>.

^3^World Health Organization. [20 March 2023]. Available from: <https://covid19.who.int/who-data/vaccination-data.csv>


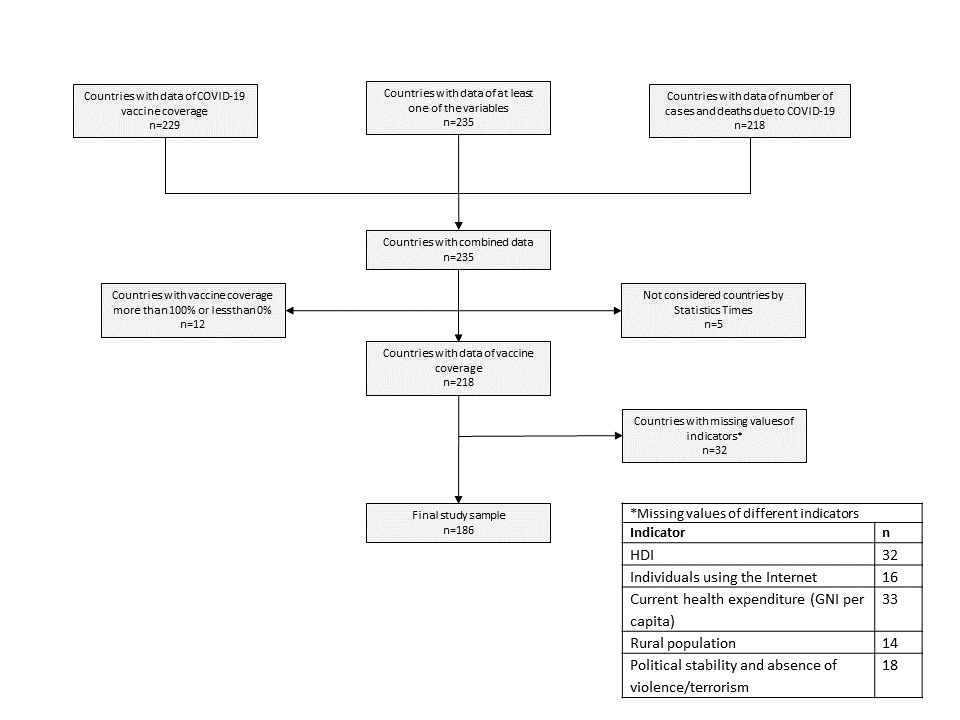


**Supplemental Figure 1. Flowchart of country sample selection (**n = number of countries)**


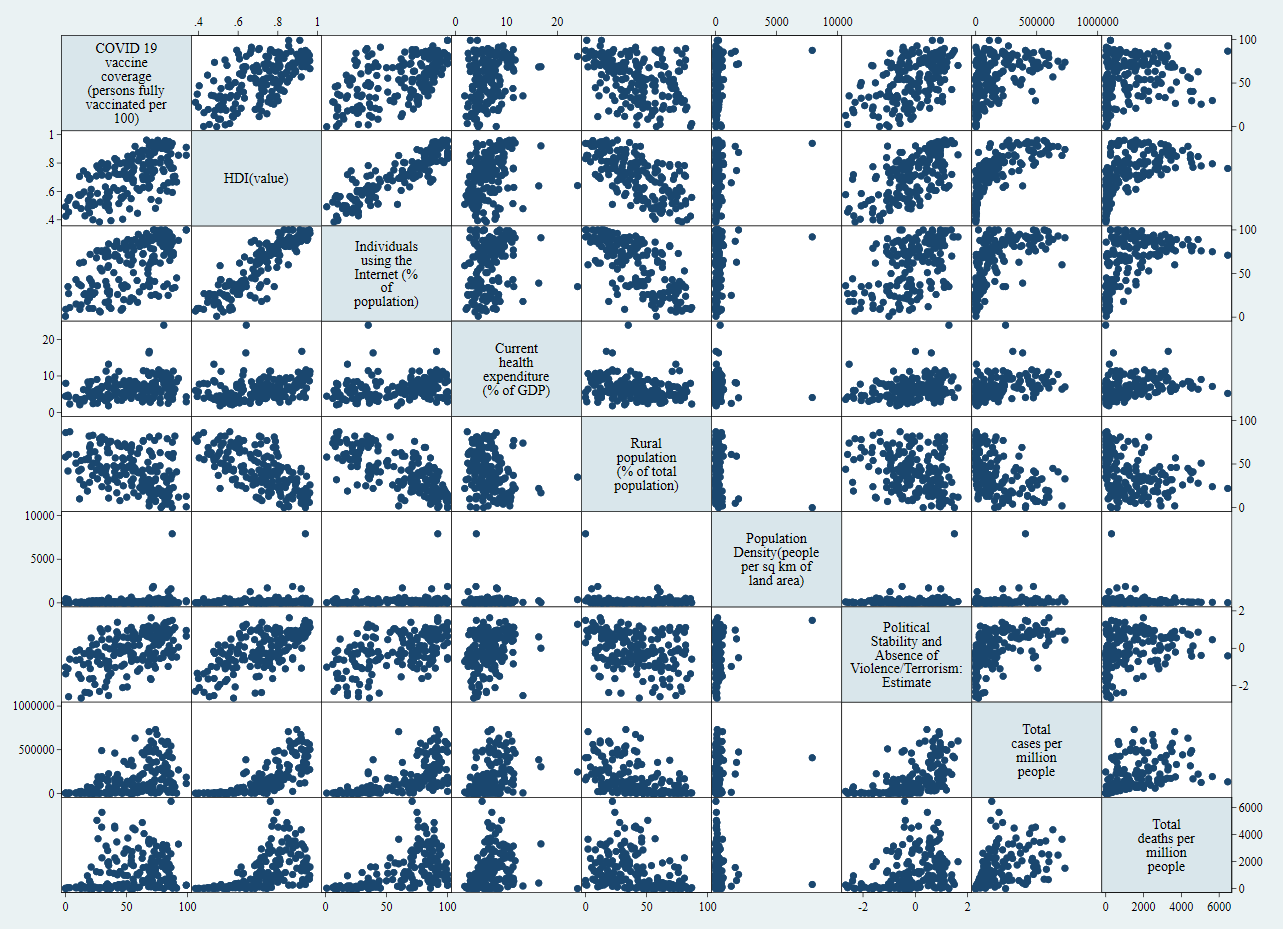


**Supplemental Figure 2. Correlation matrix of all variables (n=186)**

**
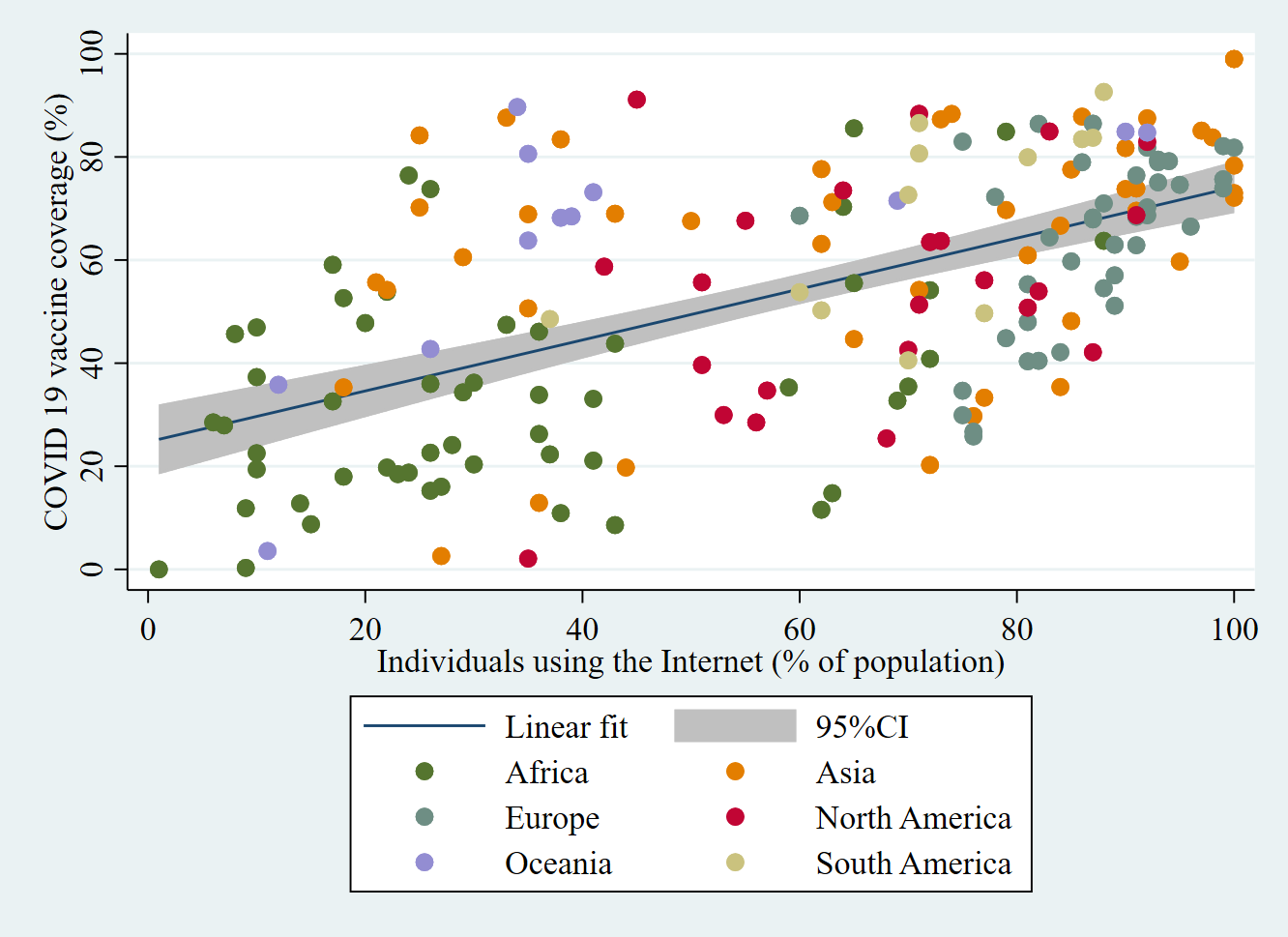
**

**Supplemental Figure 3. Correlation matrix of individuals using the internet**

**
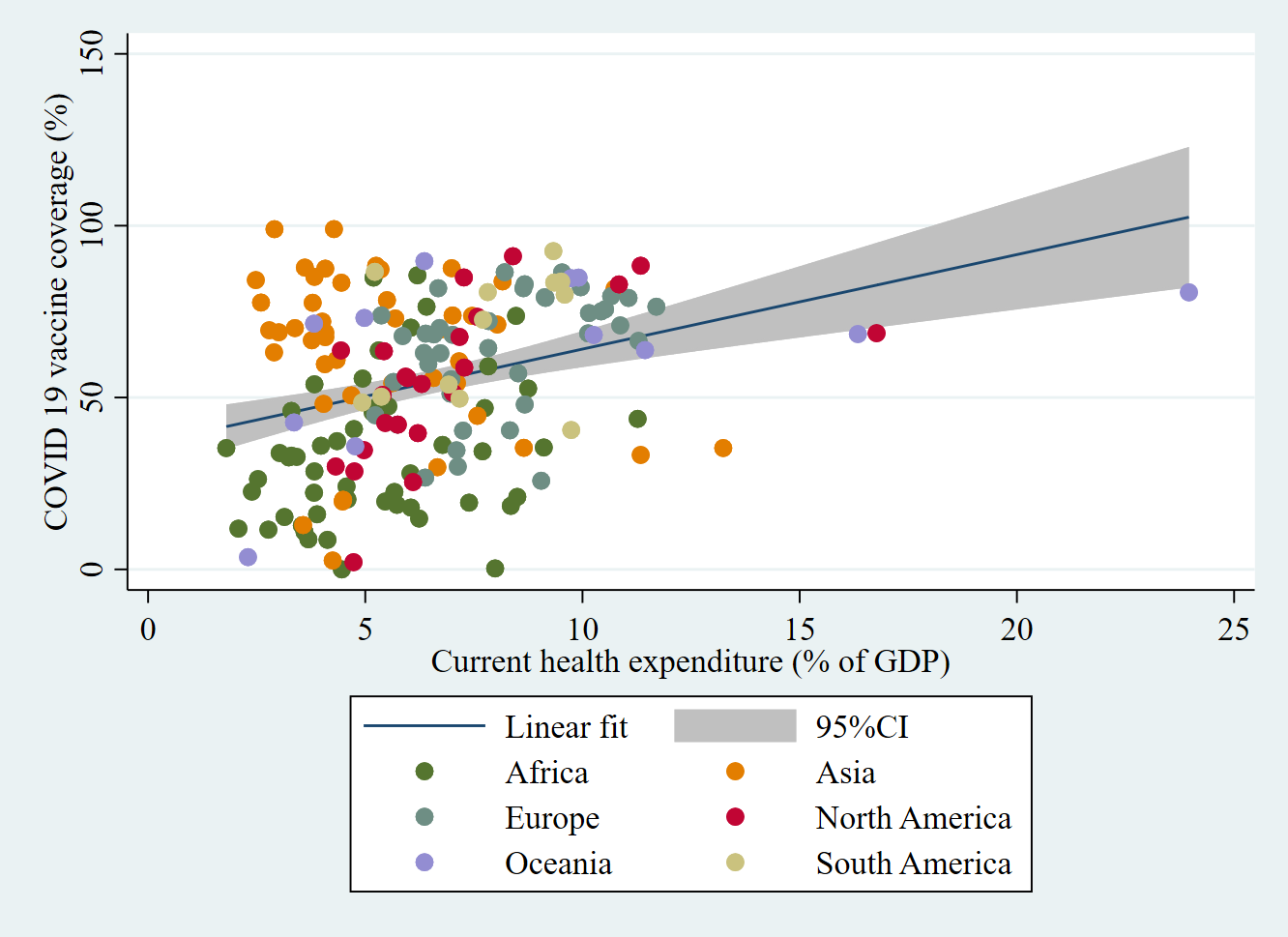
**

**Supplemental Figure 4. Correlation matrix of current health expenditure**

**
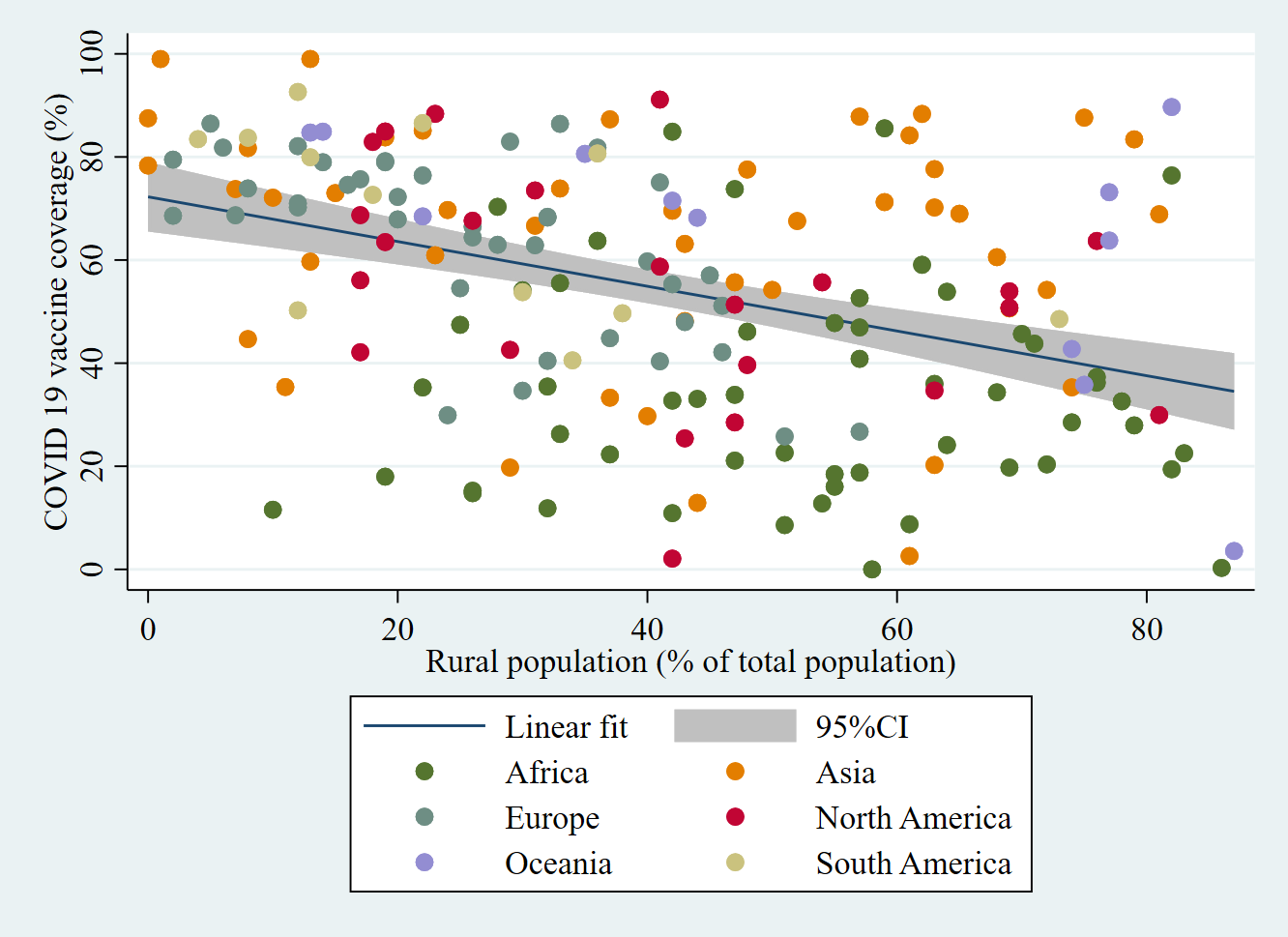
**

**Supplemental Figure 5. Correlation matrix of rural population (% of total population)**

**
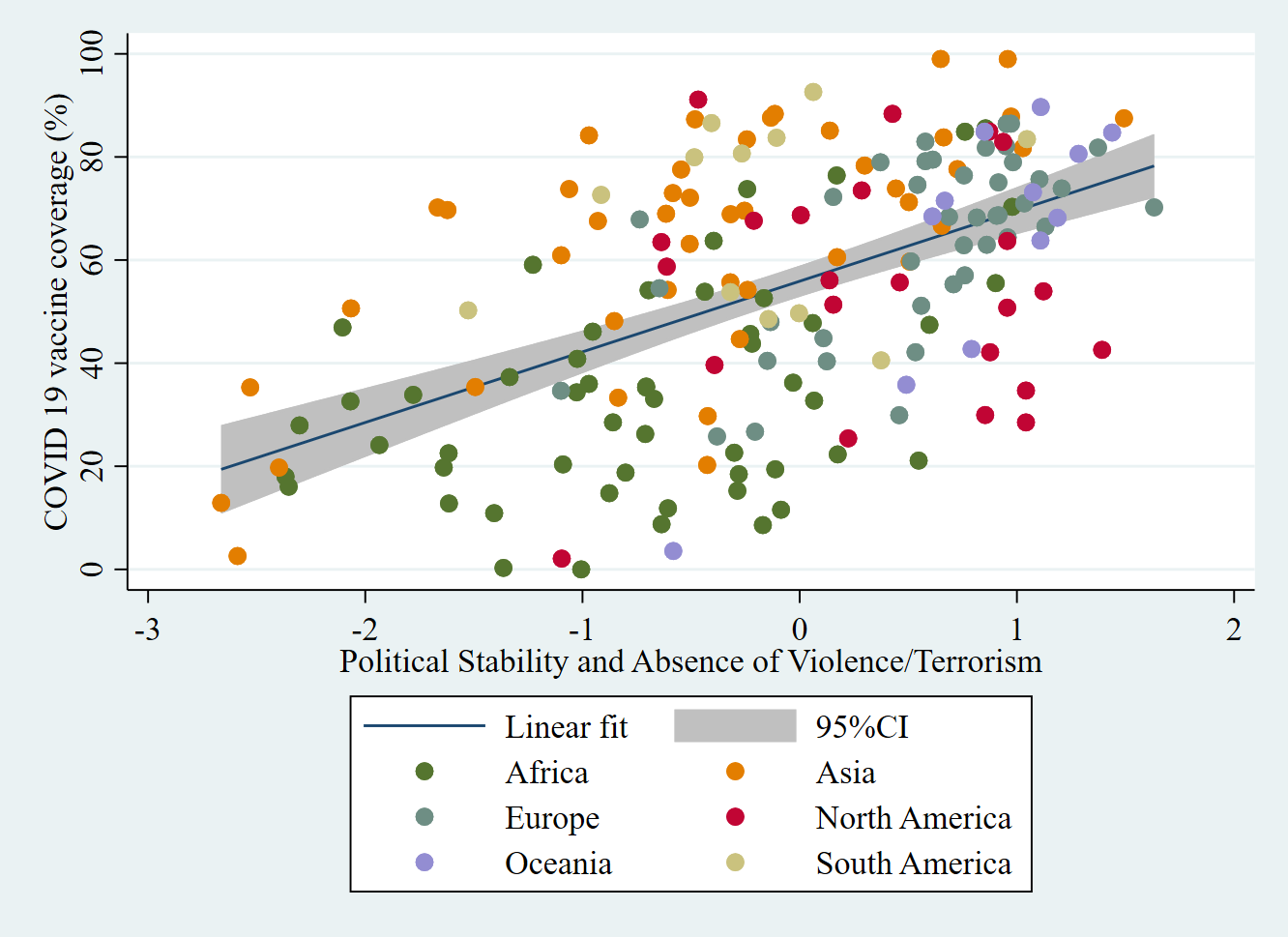
**

**Supplemental Figure 6. Correlation matrix of political stability and absence of violence/terrorism**

**
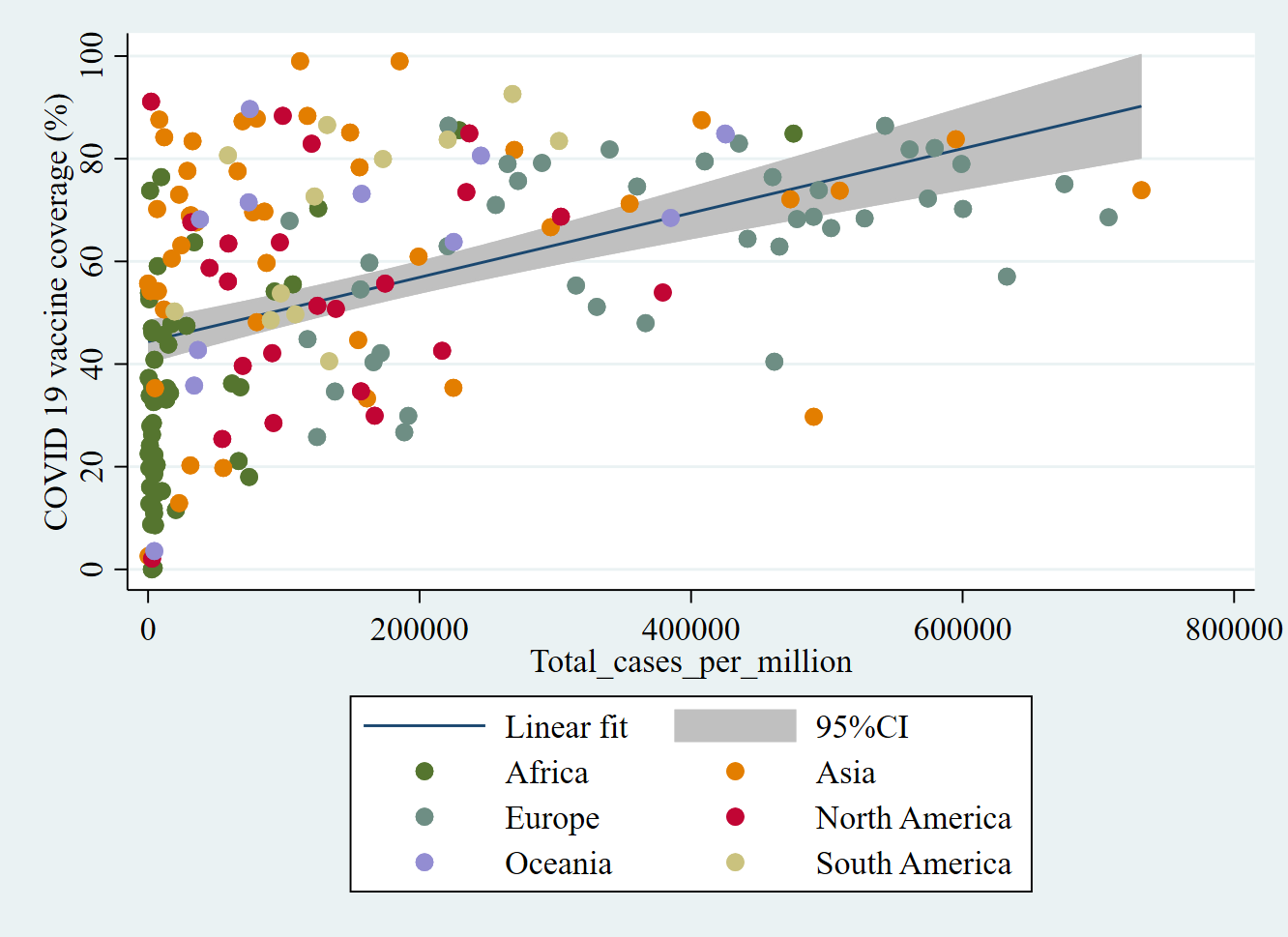
**

**Supplemental Figure 7. Correlation matrix of total cases per million**

**
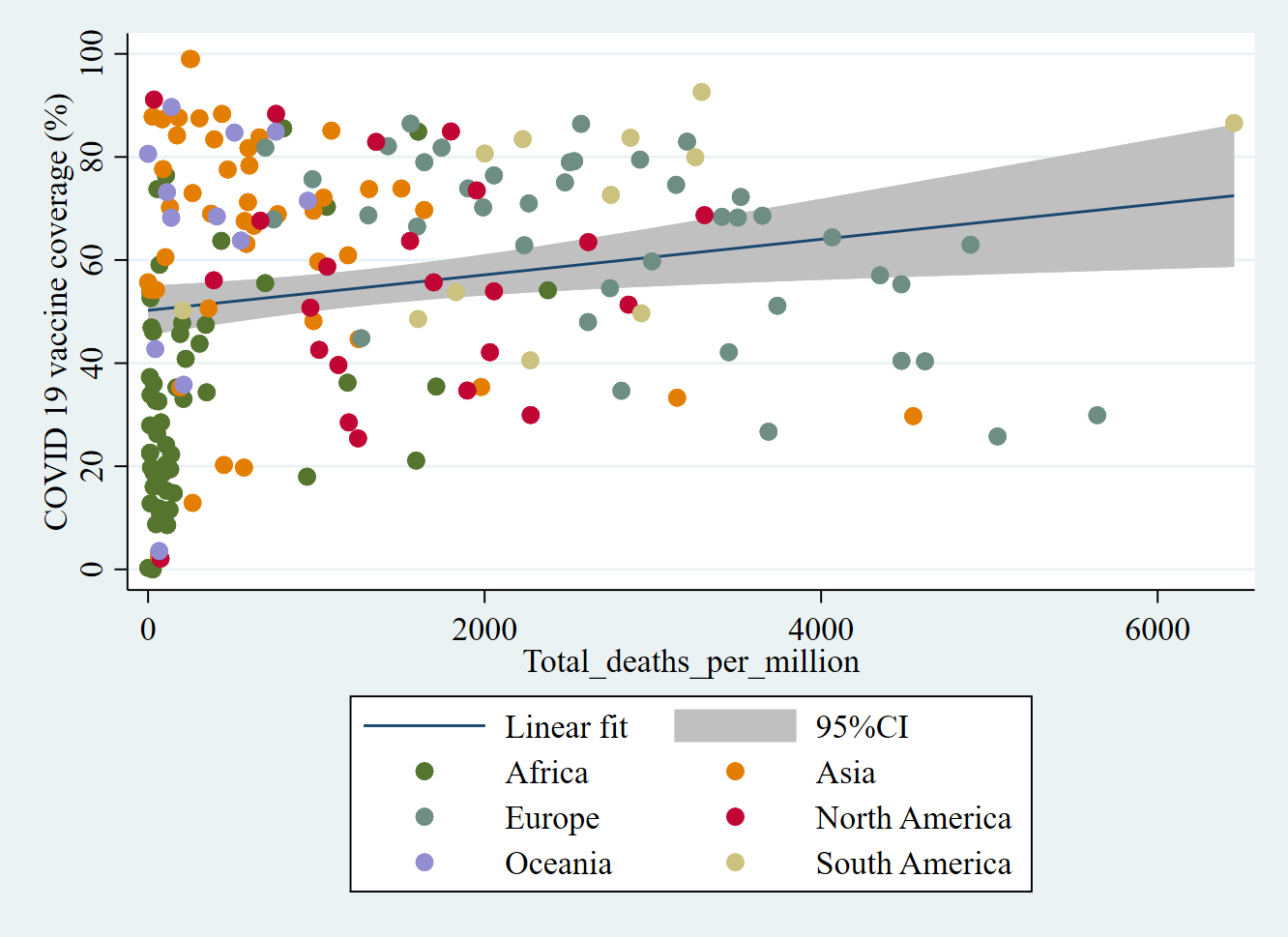
**

**Supplemental Figure 8. Correlation matrix of total deaths per million**
